# Evaluation of Tobramycin and Ciprofloxacin as a Synergistic Combination Against Hypermutable *Pseudomonas Aeruginosa* Strains via Mechanism-Based Modelling

**DOI:** 10.3390/pharmaceutics11090470

**Published:** 2019-09-12

**Authors:** Vanessa E. Rees, Jürgen B. Bulitta, Antonio Oliver, Roger L. Nation, Cornelia B. Landersdorfer

**Affiliations:** 1 Centre for Medicine Use and Safety, Monash Institute of Pharmaceutical Sciences, Monash University (Parkville campus), Parkville, VIC 3052, Australia; 2 Drug Delivery, Disposition and Dynamics, Monash Institute of Pharmaceutical Sciences, Monash University (Parkville campus), Parkville, VIC 3052, Australia; roger.nation@monash.edu; 3 The Novo Nordisk Centre for Biosustainability, The Technical University of Denmark (DTU), 2800 Kgs. Lyngby, Denmark; 4 Department of Pharmacotherapy and Translational Research, College of Pharmacy, University of Florida, Orlando, FL 32827, USA; jbulitta@cop.ufl.edu; 5 Servicio de Microbiología, Hospital Universitario Son Espases, Instituto de Investigación Sanitaria de Palma, 07120 Palma de Mallorca, Spain; antonio.oliver@ssib.es

**Keywords:** hypermutable, antipseudomonals, antibiotic resistance, antibiotic combination, mechanism-based modelling

## Abstract

Hypermutable *Pseudomonas aeruginosa* strains have a greatly increased mutation rate and are prevalent in chronic respiratory infections. Initially, we systematically evaluated the time-course of total and resistant populations of hypermutable (PAO∆*mutS*) and non-hypermutable (PAO1) *P. aeruginosa* strains in 48-h static concentration time-kill studies with two inocula. Both strains were exposed to clinically relevant concentrations of important antibiotics (aztreonam, ceftazidime, imipenem, meropenem, tobramycin, and ciprofloxacin) in monotherapy. The combination of tobramycin and ciprofloxacin was subsequently assessed in 48-h static concentration time-kill studies against PAO1, PAO∆*mutS*, and two hypermutable clinical *P. aeruginosa* strains. Mechanism-based mathematical modelling was conducted to describe the time-course of total and resistant bacteria for all four strains exposed to the combination. With all monotherapies, bacterial regrowth and resistant populations were overall more pronounced for PAO∆*mutS* compared to PAO1. The combination of tobramycin and ciprofloxacin was synergistic, with up to 10^6.1^ colony forming units (CFU)/mL more bacterial killing than the most active monotherapy for all strains, and largely suppressed less-susceptible populations. This work indicates that monotherapies against hypermutable *P. aeruginosa* strains are not a viable option. Tobramycin with ciprofloxacin was identified as a promising and tangible option to combat hypermutable *P. aeruginosa* strains.

## 1. Introduction

Respiratory infections caused by *Pseudomonas aeruginosa* are a major cause of morbidity and mortality among patients with cystic fibrosis (CF) [1]. Furthermore, hypermutable *P. aeruginosa* strains are prevalent in chronic respiratory infections of CF patients [2,3]. These hypermutable strains often result from altered or defective genes within the mismatch repair (MMR) system, which lead to an increased mutation rate [4]. As a result, hypermutable bacteria can quickly adapt to changing environments, including antibiotic exposure [3]. Suboptimal treatment of *P. aeruginosa* infections involving these hypermutable strains risks the emergence of multi-drug resistance; therefore, there is an urgent need to evaluate antibiotic therapy to improve efficacy, including suppression of the emergence of resistance [5].

Previously, minimum inhibitory concentration (MIC) values have been determined for multiple antibiotics against a hypermutable strain [6,7]. However, MICs are based on only one time-point, and use a low bacterial inoculum and a small volume of bacterial suspension. Static concentration time-kill assays allow a quantitative description of the time-course of antibacterial effects on the total and resistant bacterial populations. The impact of increased spontaneous mutation rates found in hypermutable *P. aeruginosa* on the time-course of bacterial killing, regrowth, and resistance emergence over 48 h has not been systematically evaluated for a range of antibiotics from different classes. Hence, we aimed to quantify the differences in antibacterial effects and emergence of resistant populations between hypermutable and non-hypermutable *P. aeruginosa* for the most important clinically used antipseudomonal antibiotics. Furthermore, we aimed to assess the synergistic properties and suppression of resistant populations by the combination of two fast-acting antibiotics with different mechanisms of action, tobramycin and ciprofloxacin, against hypermutable and non-hypermutable *P. aeruginosa*, including two clinical hypermutable strains. The combination had not been previously evaluated against clinical hypermutable *P. aeruginosa*. The time-courses of the total and resistant populations after exposure to this antibiotic combination were evaluated in 48-h static concentration time-kill experiments and subsequently described by mechanism-based mathematical modelling (MBM). This study indicates that monotherapies are not a viable treatment option against hypermutable *P. aeruginosa*. The rapid-acting combination of tobramycin with ciprofloxacin was found to be a promising synergistic option to combat hypermutable *P. aeruginosa*.

## 2. Materials and Methods

### 2.1. Bacterial Strains and Antibiotics Tested

We used a well-characterised hypermutable PAO∆*mutS* strain [8] and its non-hypermutable PAO1 wild-type parental strain [9] (i.e., differing only in the knockout of the *mutS* gene from the MMR system), as well as two clinical hypermutable strains (mucoid CW19 and nonmucoid CW44) obtained from respiratory infections of patients with CF [10], in 48-h static concentration time-kill studies. Bacterial killing and emergence of resistant populations were quantified for six common antipseudomonals against PAO∆*mutS* and PAO1, as follows: aztreonam (Sigma-Aldrich, Castle Hill, Australia), ceftazidime (Chem-Impex, Wood Dale, IL, USA), imipenem (MSD, Macquarie Park, Australia), meropenem (DBL Hospira, Melbourne, Australia), tobramycin (AK Scientific, Union City, CA, USA), and ciprofloxacin (Waterstone Technology, Carmel, IN, USA). All antibiotic solutions were prepared in MilliQ^®^ water and were filter-sterilised using a 0.22-µm polyvinylidene difluoride (PVDF) syringe filter (Merck Millipore, Cork, Ireland). The combination of tobramycin with ciprofloxacin was evaluated in 48-h static concentration time-kill studies and described by MBM for all four strains. The MIC values for all strains are shown in Table 1.

### 2.2. Static Concentration Time-Kill Assays

Inocula of approximately 10^5.2^ and 10^7.5^ colony forming units (CFU)/mL for monotherapy and approximately 10^7.5^ CFU/mL for combination studies were targeted. Targeted inocula were achieved in 15 mL of cation-adjusted Mueller Hinton broth (CAMHB; containing 25 mg/L Ca^2+^ and 12.5 mg/L Mg^2+^; BD, North Ryde, Australia), and static concentration time-kill studies were conducted for 48 h, as described previously [12]. In monotherapy against the PAO∆*mutS* and PAO1 strains we studied: aztreonam 1, 2, 4, 8, 16, 32, 64 and 128 mg/L; ceftazidime 1, 2, 4, 8, 32, and 64 mg/L; imipenem 0.5, 1, 2, 4, 16, and 64 mg/L; meropenem 0.25, 0.5, 1, 2, 8, and 32 mg/L; tobramycin 0.25, 0.5, 1, 2, 4, 8, 16, and 32 mg/L; and ciprofloxacin 0.0625, 0.125, 0.25, 0.5, 1, 2, and 4 mg/L. At 24 h, 100% re-dosing after a centrifugation and re-suspension step was performed for aztreonam, imipenem, and meropenem; the carbapenems also had an additional 30% (meropenem) or 50% (imipenem) supplementation at 6 and 30 h to offset thermal degradation [13,14]. Biological replicate static concentration time-kill studies (*n* = 2–4) were performed for an antibiotic from each class at a clinically relevant concentration against the high inoculum of strains PAO∆*mutS* and PAO1. The effect of combining two rapidly killing antibiotics was examined using tobramycin (1, 4 and 8 mg/L) and ciprofloxacin (0.125, 1 and 4 mg/L) in monotherapy and combination therapy against PAO∆*mutS* and PAO1 strains and the two clinical strains (CW19 and CW44). The approximate unbound average steady-state plasma concentration of the maximum daily dose for each antibiotic can be found in Table S1.

### 2.3. Viable Counting of the Total and Resistant Populations

Viability counts were determined at 0 (5 min prior to dosing), 1.5, 3, 6, 24, 29 (excluding aztreonam 10^7.5^ CFU/mL and ceftazidime at both inocula) and 48 h, as previously described [12], to establish the time-course of the total population on drug-free cation-adjusted Mueller Hinton agar (CAMHA; containing 25 mg/L Ca^2+^ and 12.5 mg/L Mg^2+^; BD, North Ryde, Australia) and resistant populations at 24 and 48 h on antibiotic-containing CAMHA. The antibiotic concentrations used in CAMHA to quantify the resistant populations for the monotherapy studies were: 20 mg/L aztreonam, 10 mg/L ceftazidime, 10 mg/L imipenem, 5 mg/L meropenem, 2.5 mg/L tobramycin, and 1.25 mg/L ciprofloxacin. The tobramycin and ciprofloxacin combination studies with PAO1 and PAO∆*mutS* examined the less-susceptible populations on 2.5 mg/L tobramycin- and 0.625 mg/L ciprofloxacin-containing CAMHA (a lower ciprofloxacin concentration was used as the combination was anticipated to reduce the emergence of resistant populations). For CW19 the concentrations in agar were 2.5 mg/L ciprofloxacin and 5 mg/L tobramycin, and for CW44 they were 5 mg/L ciprofloxacin and 2.5 mg/L tobramycin. The limit of counting was 1.0 log_10_ CFU/mL on antibiotic-free agar plates and 0.7 log_10_ CFU/mL on antibiotic-containing agar plates. Samples with no detectable colonies were plotted at 0 log_10_ CFU/mL. The significance of differences between strains PAO∆*mutS* and PAO1 for biological replicates was assessed using independent *t*-tests.

### 2.4. Mechanism-Based Modelling of Bacterial Killing and Resistance

MBM was conducted to quantitatively characterise the time-course of bacterial killing and any regrowth of the total and less-susceptible *P. aeruginosa* populations against tobramycin and ciprofloxacin alone and in combination. We used S-ADAPT (version 1.57) facilitated by SADAPT-TRAN with the importance sampling algorithm (pmethod = 4) [15]. The between-curve variability of the parameters was set to a coefficient of variation of 15% during the end of the estimation [16]. Competing models were evaluated based on the visual predictive checks, standard diagnostic plots, S-ADAPT objective function value (−1 × log-likelihood), biological plausibility of the parameter estimates, and the coefficient of correlation [17,18,19].

#### 2.4.1. The Life-Cycle Growth Model

**Figure 1:**
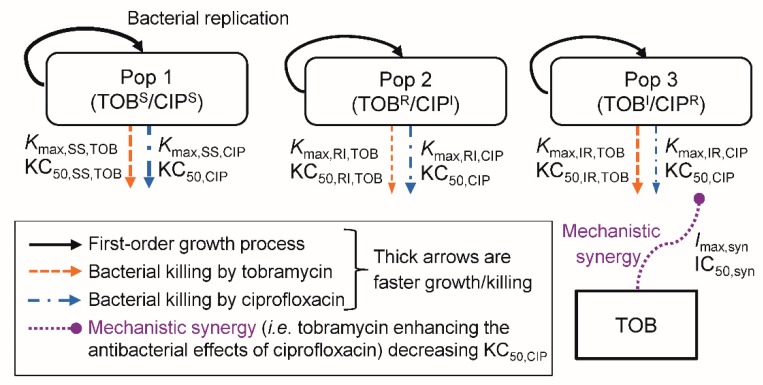
The mechanism-based model for bacterial growth and killing by tobramycin and ciprofloxacin. The TOB^S^/CIP^S^ population is susceptible to both antibiotics, the TOB^R^/CIP^I^ population is tobramycin-resistant and ciprofloxacin-intermediate, and the TOB^I^/CIP^R^ population is tobramycin-intermediate and ciprofloxacin-resistant. The underlying biological mechanisms of bacterial replication are described by a life-cycle growth model. All parameters, including the maximum killing rate constants (*K_max_*), the related concentrations of antibiotic causing 50% of *K_max_* (KC_50_), and the mechanistic synergy terms (*I_max,syn_* and IC_50,syn_) are displayed in Table 2.

The life-cycle growth model that accounts for underlying biological processes was used to describe the growth and replication of *P. aeruginosa* [20,21,22]. The proposed model (Figure 1) for the combination of tobramycin and ciprofloxacin was comprised of three pre-existing bacterial subpopulations; double-susceptible (SS), tobramycin-resistant and ciprofloxacin-intermediate (RI), and tobramycin-intermediate and ciprofloxacin-resistant (IR) populations. Two bacterial states (i.e., compartments) were described for each of these subpopulations: state 1 including bacteria preparing for replication and state 2 for bacteria immediately before replication [21,22,23,24]. Thus, for the double-susceptible population, CFU_SS1_ represented the bacteria in state 1 and CFU_SS2_ the bacteria in state 2. Correspondingly, CFU_RI1_ and CFU_RI2_ described the tobramycin-resistant and ciprofloxacin-intermediate population, and CFU_IR1_ and CFU_IR2_ the tobramycin-intermediate and ciprofloxacin-resistant population.

The total bacterial population (CFU_All_) was defined as the sum of bacteria in all bacterial subpopulations in both states:

(1) $${\mathrm{CFU}}_{\mathrm{all}}={\mathrm{CFU}}_{\mathrm{SS}1}+{\mathrm{CFU}}_{\mathrm{SS}2}+{\mathrm{CFU}}_{\mathrm{RI}1}+{\mathrm{CFU}}_{\mathrm{RI}2}+{\mathrm{CFU}}_{\mathrm{IR}1}+{\mathrm{CFU}}_{\mathrm{IR}2}$$

CFU_IR1_ was described by:

(2) $$\begin{array}{ll} \frac{d({\mathrm{CFU}}_{\mathrm{IR}1})}{dt}= & 2\cdot \mathrm{PLAT}\cdot k_{21}\cdot {\mathrm{CFU}}_{\mathrm{IR}2}-k_{12,\mathrm{IR}}\cdot {\mathrm{CFU}}_{\mathrm{IR}1}\\ & -\left(\frac{K_{\max,\mathrm{IR},\mathrm{TOB}\;}\cdot {\;C}_{\mathrm{TOB}}{}^{{\mathrm{Hill}}_{\mathrm{TOB}}}}{C_{\mathrm{TOB}}{}^{{\mathrm{Hill}}_{\mathrm{TOB}}}+{\mathrm{KC}}_{50,\mathrm{TOB}}{}^{{\mathrm{Hill}}_{\mathrm{TOB}}}}+\frac{K_{\max,\mathrm{IR},\mathrm{CIP}\;}\cdot {\;C}_{\mathrm{CIP}}}{C_{\mathrm{CIP}}+\;\left(\mathrm{SYN}\;\cdot {\;\mathrm{KC}}_{50,\mathrm{CIP}}\right)}\right)\cdot {\mathrm{CFU}}_{\mathrm{IR}1} \end{array}$$

where the factor 2 represented the doubling of bacteria during replication. The plateau factor (PLAT) described the probability of successful replication and was defined as 1 – (CFU_all_/(CFU_all_ + CFU_max_)), with CFU_max_ being the maximum population size [21,25,26]. The first-order replication rate constant $\left(k_{21}\right)$ was set to 50 h^−1^ as replication is rapid [22]. The first-order growth rate constant $\left(k_{12,\mathrm{IR}}\right)$ was defined as 60/MGT_IR_, with MGT_IR_ denoting the mean generation time for the bacterial population. We used a direct killing process [25,26,27] for both tobramycin and ciprofloxacin. The KC_50,TOB_ and KC_50,CIP_ were the tobramycin and ciprofloxacin concentrations required to achieve 50% of the maximum killing rate constant (*K*_max_). The C_TOB_ and C_CIP_ were the concentrations of tobramycin and ciprofloxacin in broth, and Hill_TOB_ was the Hill coefficient for tobramycin (only required for PAO1). The term SYN (i.e., mechanistic synergy) is described in Equation (4). CFU_IR2_ was described by:

(3) $$\begin{array}{ll} \frac{d({\mathrm{CFU}}_{\mathrm{IR}2})}{dt}= & -k_{21}\cdot {\mathrm{CFU}}_{\mathrm{IR}2}+k_{12,\mathrm{IR}}\cdot {\mathrm{CFU}}_{\mathrm{IR}1}\\ & -\left(\frac{K_{\max,\mathrm{IR},\mathrm{TOB}\;}\cdot {\;C}_{\mathrm{TOB}}{}^{{\mathrm{Hill}}_{\mathrm{TOB}}}}{C_{\mathrm{TOB}}{}^{{\mathrm{Hill}}_{\mathrm{TOB}}}+{\mathrm{KC}}_{50,\mathrm{TOB}}{}^{{\mathrm{Hill}}_{\mathrm{TOB}}}}+\frac{K_{\max,\mathrm{IR},\mathrm{CIP}\;}\cdot {\;C}_{\mathrm{CIP}}}{C_{\mathrm{CIP}}+\;\left(\mathrm{SYN}\;\cdot {\;\mathrm{KC}}_{50,\mathrm{CIP}}\right)}\right)\cdot {\mathrm{CFU}}_{\mathrm{IR}2} \end{array}$$

The double-susceptible and the tobramycin-resistant and ciprofloxacin-intermediate populations were modelled similarly, except they had different estimates for *K*_max_ and *k*_12_ compared to the tobramycin-intermediate and ciprofloxacin-resistant population.

#### 2.4.2. Synergy Modelling

We evaluated subpopulation synergy (i.e., tobramycin killing the bacteria resistant to ciprofloxacin and vice versa) and mechanistic synergy (i.e., tobramycin enhancing the bacterial killing by ciprofloxacin) [21,25,26,27]. The mechanistic synergy equation was:

(4) $$\mathrm{SYN}=1-\left(\frac{I_{\max,\mathrm{SYN}}\;\cdot {\;C}_{\mathrm{TOB}}}{C_{\mathrm{TOB}}+{\;\mathrm{IC}}_{50,\mathrm{SYN}}}\right)$$

where *I*_max,SYN_ was the maximum fractional decrease of KC_50,CIP_ due to mechanistic synergy, and IC_50,SYN_ was the tobramycin concentration causing 50% of *I*_max,SYN_.

#### 2.4.3. Less-Susceptible Bacterial Populations

The viable counts on tobramycin-containing agar and ciprofloxacin-containing agar were modelled simultaneously with the total viable counts on drug-free agar. The fractions of subpopulations (susceptible, intermediate, resistant) that were able to grow on tobramycin- and ciprofloxacin-containing agar plates at different concentrations were estimated as described previously [28,29].

#### 2.4.4. Initial Conditions and Observation Model

The total initial inocula (Log_10_ CFU_0_) and the mutation frequencies for the tobramycin-resistant and ciprofloxacin-intermediate (Log_10_ MF_TOB_), and tobramycin-intermediate and ciprofloxacin-resistant (Log_10_ MF_CIP_) populations were estimated (Table 2). The susceptible bacterial population was calculated by subtracting the initial conditions of the intermediate and the resistant populations from the respective total inoculum. Bacteria were initialised in state 1 and the initial conditions for CFU_SS2_, CFU_RI2_ and CFU_IR2_ were set to zero. An additive residual error model was used to fit the viability counts on log_10_ scale. A previously developed residual error model was used to directly fit the number of colonies per plate for observations below 2 log_10_ CFU/mL [16].

## 3. Results and Discussion

### 3.1. Antibacterial Effect of Common Antipseudomonal Antibiotics in Monotherapy vs PAO1 and PAO∆mutS

**Figure 2:**
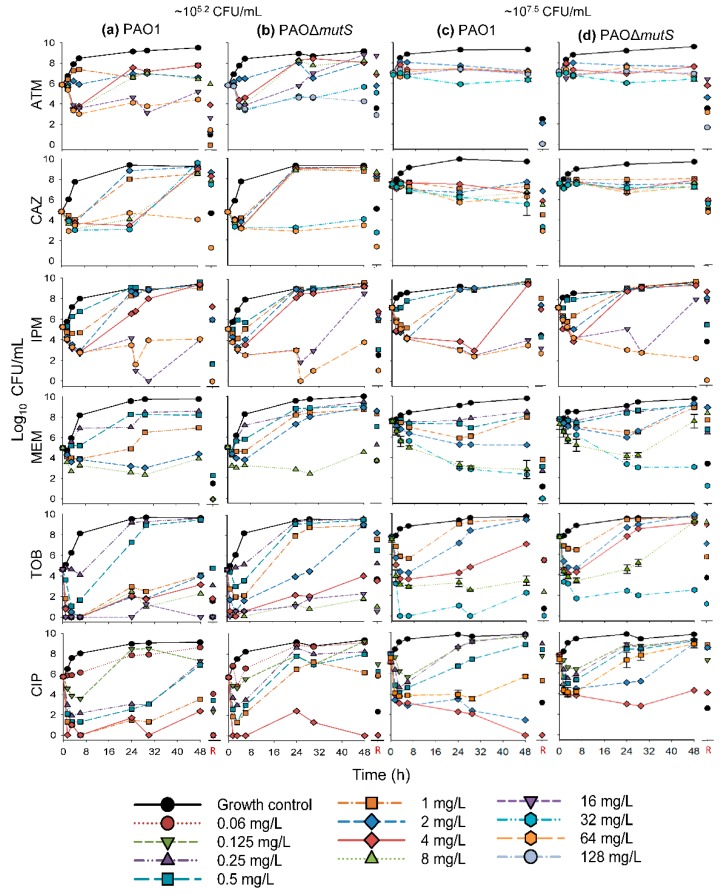
The log_10_ viability counts (CFU/mL) of bacterial growth on antibiotic-free agar plates (total populations) versus time for non-hypermutable PAO1 and hypermutable PAO∆*mutS* exposed to aztreonam (ATM), ceftazidime (CAZ), imipenem (IPM), meropenem (MEM), tobramycin (TOB), and ciprofloxacin (CIP) in 48-h static concentration time-kill experiments at two initial inocula (lower ~10^5.2^ CFU/mL on the left, (**a**) PAO1, (**b**) PAO∆*mutS*,and higher ~10^7.5^ CFU/mL on the right, (**c**) PAO1, (**d**) PAO∆*mutS*). The following concentrations were studied against both inocula of each strain (unless denoted otherwise): aztreonam 1^a^, 2, 4, 8^b^, 16, 32^c,d^, 64^a,d^, and 128^c,d^ mg/L; ceftazidime 1, 2, 4, 8, 32, and 64 mg/L; imipenem 0.5, 1, 2, 4, 16, and 64 mg/L; meropenem 0.25, 0.5, 1, 2, 8, and 32^d^ mg/L; tobramycin 0.25^b^, 0.5^b^, 1, 2, 4, 8, 16^b^, and 32^d^ mg/L; and ciprofloxacin 0.0625^b^, 0.125, 0.25, 0.5, 1, 2^d^, and 4 mg/L. The biological replicate studies for meropenem (8 mg/L), tobramycin (8 mg/L), and ciprofloxacin (1 mg/L) revealed significant differences (*p* < 0.001, *p* < 0.01 and *p* < 0.001, respectively) between the two strains in the bacterial density at 48 h; see also Figure S1. R: denotes the bacterial population that grew on antibiotic-containing agar plates at 48 h. ^a^ Concentration was only used against the lower inoculum (10^5.2^ CFU/mL) of PAO1. ^b^ Concentration was only used against the lower inoculum (10^5.2^ CFU/mL) of both strains. ^c^ Concentration was only used against the lower inoculum (10^5.2^ CFU/mL) of PAO∆*mutS*. ^d^ Concentration was only used against the higher inoculum (10^7.5^ CFU/mL) of both strains.

**Figure 3:**
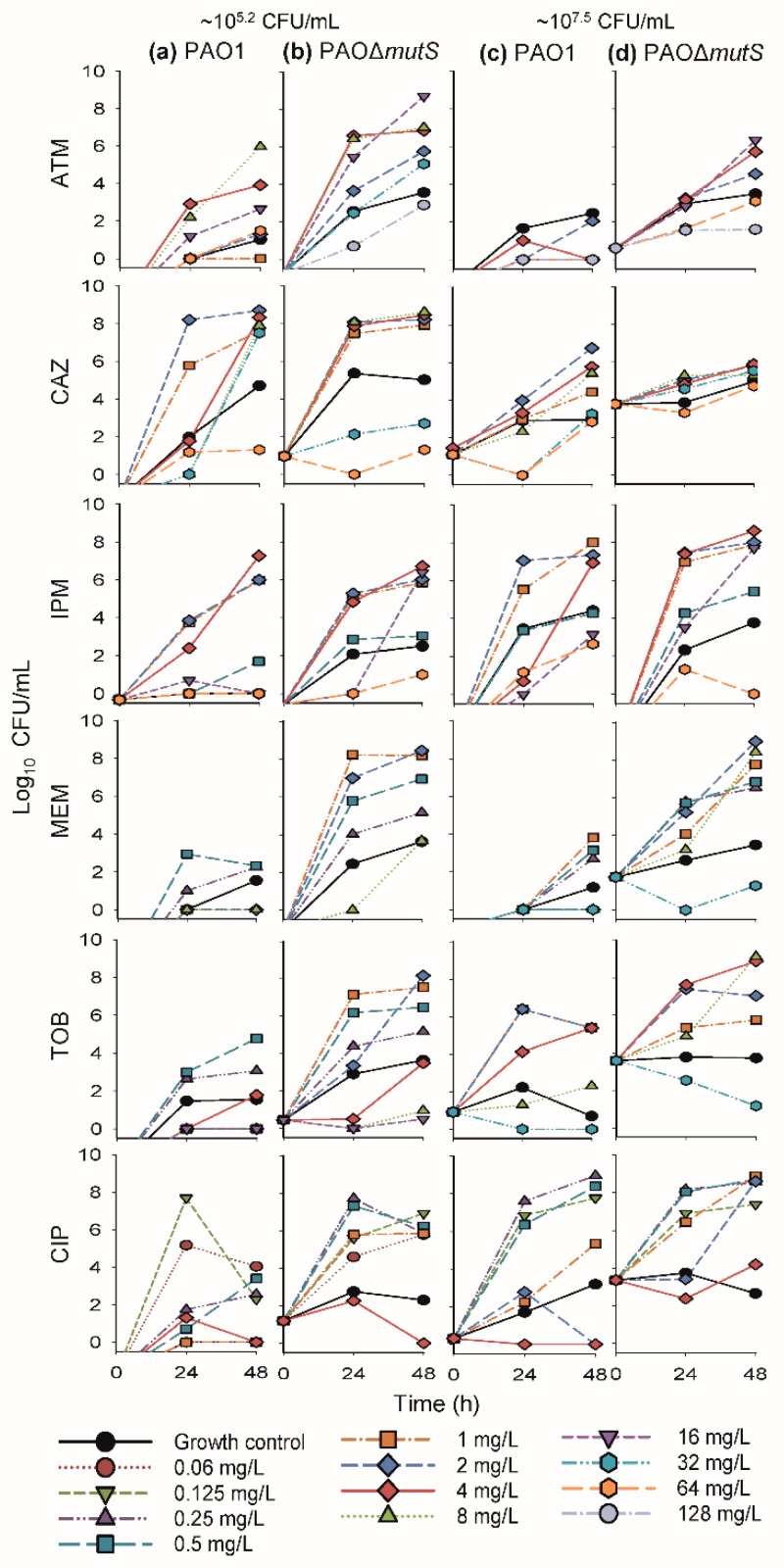
The log_10_ viability counts (CFU/mL) of bacterial growth on antibiotic-containing agar plates (resistant populations) versus time for non-hypermutable PAO1 and hypermutable PAO∆*mutS* in 48-h static concentration time-kill experiments at two initial inocula (lower ~10^5.2^ CFU/mL on the left, (**a**) PAO1, (**b**) PAO∆*mutS*, and higher ~10^7.5^ CFU/mL, on the right, (**c**) PAO1, (**d**) PAO∆*mutS*). Antibiotic concentrations in agar were: 20 mg/L aztreonam (ATM), 10 mg/L ceftazidime (CAZ), 10 mg/L imipenem (IPM), 5 mg/L meropenem (MEM), 2.5 mg/L tobramycin (TOB), and 1.25 mg/L ciprofloxacin (CIP). The following concentrations were studied against both inocula of each strain (unless denoted otherwise): aztreonam 1^a^, 2, 4, 8^b^, 16, 32^c^, 64^a,d^, and 128^c,d^ mg/L; ceftazidime 1, 2, 4, 8, 32, and 64 mg/L; imipenem 0.5, 1, 2, 4, 16, and 64 mg/L; meropenem 0.25, 0.5, 1, 2, 8, and 32^d^ mg/L; tobramycin 0.25^b^, 0.5^b^, 1, 2, 4, 8, 16^b^, and 32^d^ mg/L; and ciprofloxacin 0.0625^b^, 0.125, 0.25, 0.5, 1, 2^d^, and 4 mg/L. ^a^ Concentration was only used against the lower inoculum (10^5.2^ CFU/mL) of PAO1. ^b^ Concentration was only used against the lower inoculum (10^5.2^ CFU/mL) of both strains. ^c^ Concentration was only used against the lower inoculum (10^5.2^ CFU/mL) of PAO∆*mutS*. ^d^ Concentration was only used against the higher inoculum (10^7.5^ CFU/mL) of both strains.

The time-course profiles of bacterial density as determined on antibiotic-free plates (i.e., total bacterial count) and antibiotic-containing plates (i.e., resistant subpopulations) for the monotherapy investigation are presented in Figure 2 and Figure 3, respectively. Additionally, the results of the biological replicate studies are presented in Figure S1. The resistant populations of the growth controls (i.e., absence of antibiotic during the time-course studies) were generally larger for the hypermutable PAO∆*mutS* than the non-hypermutable PAO1 (Figure 3). In the presence of antibiotic during the time-course studies, overall there was less bacterial killing and suppression of resistant populations of PAO∆*mutS* compared to the PAO1 strain. In contrast to the minimal yield of information conveyed in an MIC estimate [30], the collection of multiple samples over 48 h in the present study allowed us to evaluate the antibacterial effects on not only killing but also regrowth and emergence of resistant populations. Our two studied inocula were intended to simulate infections with different bacterial densities.

#### 3.1.1. Antibacterial Effect of Beta-Lactam Antibiotics in Monotherapy

The β-lactam aztreonam showed differences in bacterial regrowth (Figure 2) and emergence of resistant populations (Figure 3) between the two strains at the lower inoculum. Resistant bacteria had completely replaced the susceptible bacteria by 48 h when PAO∆*mutS* was exposed to 16 mg/L aztreonam. Similarly, for the lower inoculum with exposure to 32 mg/L aztreonam (equivalent to the unbound average steady-state plasma concentration for the maximum daily dose [31]), almost the whole population of PAO∆*mutS* at 48 h (~5.7 log_10_ CFU/mL) was replaced by resistant bacteria. However, PAO1 showed a reduced extent of aztreonam-resistant populations compared to PAO∆*mutS*. The β-lactam ceftazidime against the lower inoculum had earlier bacterial regrowth of PAO∆*mutS* compared to PAO1 (Figure 2). While the size of the resistant population at 48 h was comparable between the strains with both inocula, higher resistant bacterial counts were generally observed for the PAO∆*mutS* compared to PAO1 at 24 h (Figure 3). For both ceftazidime and aztreonam a pronounced inoculum effect (very limited antibacterial effect at the higher inoculum) was observed for both strains (Figure 2), as described previously for non-hypermutable *P. aeruginosa* [22,32].

The carbapenem imipenem had mostly comparable antibacterial effects between the strains with both inocula (Figure 2 and Figure 3), but some differences did occur. A 4× higher imipenem concentration (64 mg/L that is not clinically achievable vs. clinically achievable 16 mg/L [31]) was required to largely suppress regrowth and resistant populations of PAO∆*mutS* compared to PAO1. At the lower inoculum a resistant population of ~6.4 log_10_ CFU/mL for PAO∆*mutS*, and none for PAO1, was found at 48 h against the 16 mg/L imipenem (equivalent to the unbound average steady-state plasma concentration for the maximum daily dose of 4 g [31]). Furthermore, the higher inoculum against the 16 mg/L imipenem had a resistant population of PAO∆*mutS* that was ~4.5 log_10_ CFU/mL (~34,000-fold) greater than that of PAO1. Imipenem was previously assessed against a very low inoculum (~10^4.6^ log_10_ CFU/well) of PAO1 and PAO∆*mutS* in 24-h static concentration time-kill assays at only two concentrations (4 and 8 mg/L) [7]; antibacterial effects were more pronounced for PAO1 than PAO∆*mutS*.

Exposure to the carbapenem meropenem resulted in substantially greater bacterial regrowth and the emergence of less-susceptible populations for PAO∆*mutS* compared to PAO1 (Figure 2 and Figure 3). Notably, the higher inoculum against 8 mg/L meropenem (equivalent to the average steady-state plasma concentration in patients receiving the standard daily dose of 3 g [31]) led to regrowth of PAO∆*mutS* to ~7.6 log_10_ CFU/mL at 48 h with the whole population replaced by less-susceptible bacteria. In contrast, at 48 h regrowth and less-susceptible populations were largely suppressed for PAO1 compared to PAO∆*mutS* (Figure 2 and Figure S1; *p* < 0.001). Interestingly, a previous study assessed intermittent meropenem (1 g thrice daily as 30-min infusions) against PAO1 and a hypermutable clinical *P. aeruginosa* strain (from a wound swab of an intensive care unit patient) in a 24-h dynamic in vitro model; that study showed less-susceptible populations for both strains even at 24 h [33].

#### 3.1.2. Antibacterial Effect of Fast-Acting Antipseudomonal Antibiotics in Monotherapy

The aminoglycoside tobramycin initially achieved substantial bacterial killing of both strains (Figure 2). This was followed by more extensive regrowth of less-susceptible populations at 48 h for PAO∆*mutS* compared to PAO1 at both inocula (Figure 3). At the lower inoculum, a 4× higher concentration (4 vs 1 mg/L) was required to suppress regrowth to <4.0 log_10_ CFU/mL of PAO∆*mutS* compared to PAO1. For the high inoculum, tobramycin 8 mg/L suppressed regrowth and less-susceptible populations of PAO1 over 48 h; PAO∆*mutS* regrew to ~9.2 log_10_ CFU/mL at 48 h with ~9.1 log_10_ CFU/mL of less-susceptible bacteria (Figure 2 and Figure S1; *p* < 0.01). Notably, 32 mg/L tobramycin (which clinically can only be achieved for a very short time as a peak concentration [31]) was required to suppress regrowth of PAO∆*mutS*. Previously, we studied tobramycin in 24-h static concentration time-kill studies against both strains with inocula of 10^6^ and 10^4^ CFU/mL [29]; the results for the 10^6^ CFU/mL inoculum in that study were in accord with those for the lower (10^5.2^ CFU/mL) inoculum in the present study.

The fluoroquinolone ciprofloxacin displayed earlier regrowth of PAO∆*mutS* than PAO1 at both inocula (Figure 2). Ciprofloxacin 4 mg/L (which is not clinically achievable as an unbound concentration in plasma [31]) at both inocula was required to largely suppress regrowth of PAO∆*mutS*, whereas 1 mg/L was sufficient for PAO1. This was in agreement with a previous 24-h static concentration time-kill study where 1 mg/L ciprofloxacin against a very low inoculum (~4.6 log_10_ CFU/well) prevented regrowth of PAO1 whilst PAO∆*mutS* exhibited regrowth after 6 h [7]. In the present study at 48 h, the lower inoculum against 1 mg/L ciprofloxacin had a resistant population of ~5.9 log_10_ CFU/mL for PAO∆*mutS*, and none for PAO1 (Figure 3). Additionally, at 48 h the higher inoculum against 1 mg/L ciprofloxacin had ~3.1 log_10_ CFU/mL more bacterial regrowth for PAO∆*mutS* than PAO1 (Figure 2 2 and Figure S1; *p* < 0.001); the resistant population was ~3.5 log_10_ CFU/mL (~3500-fold) greater for PAO∆*mutS* than PAO1. A resistant population of ~8.6 log_10_ CFU/mL for PAO∆*mutS* (and none for PAO1) was found at the higher inoculum against 2 mg/L ciprofloxacin at 48 h.

For ciprofloxacin and tobramycin, which are clinically only administered intermittently, concentrations at or above the highest clinically achievable unbound peak plasma concentrations were required to suppress regrowth of hypermutable PAOΔ*mutS* (whilst non-hypermutable PAO1 only needed lower clinically achievable concentrations) over 48 h with inocula of 10^5.2^–10^7.5^ CFU/mL. This suggests ciprofloxacin and tobramycin in monotherapy would not be expected to be successful against hypermutable *P. aeruginosa* strains.

### 3.2. Antibacterial Effect of Two Fast-Acting Antipseudomonal Antibiotics in Combination

In view of the demonstrated inability of the antipseudomonal antibiotics in monotherapy to kill and prevent regrowth of resistant subpopulations, especially for the hypermutable PAO∆*mutS* strain, we examined a combination of the two agents that provided the greatest extent of initial killing, tobramycin and ciprofloxacin (Figure 2). The initial rapid reduction in bacterial density would be expected to decrease the likelihood of a mutation arising that confers resistance. Tobramycin and ciprofloxacin also have different mechanisms of resistance [34], which may contribute to synergy between the antibiotics. Additionally, it has been shown previously that tobramycin-resistant *P. aeruginosa* had increased susceptibility to ciprofloxacin [35]. Thus, the combination of tobramycin with ciprofloxacin was considered worthy of investigation.

**Figure 4:**
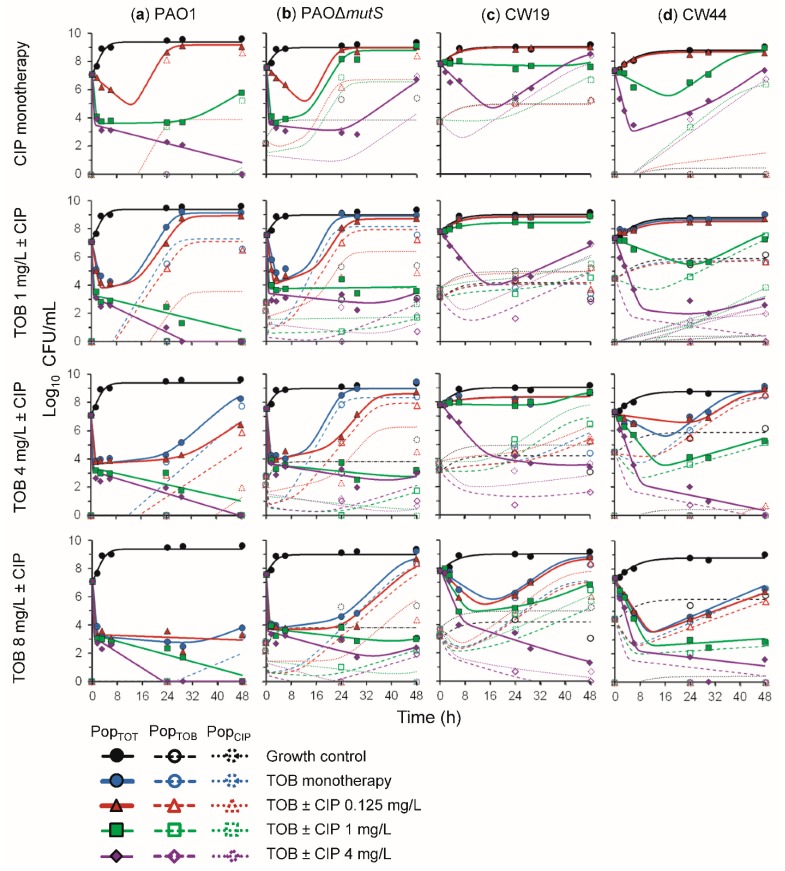
The log_10_ viability counts (CFU/mL) of bacterial growth versus time for (**a**) non-hypermutable PAO1, (**b**) hypermutable PAO∆*mutS*, (**c**) CW19, and (**d**) CW44 exposed to tobramycin (TOB) and ciprofloxacin (CIP) alone and combined in 48-h static concentration time-kill experiments at an inoculum of ~10^7.5^ CFU/mL. The top-tier panels show the growth control and ciprofloxacin monotherapies, the second-tier panels show the 1 mg/L tobramycin in monotherapy and combinations with ciprofloxacin, the third-tier panels show the 4 mg/L tobramycin in monotherapy and combinations with ciprofloxacin, and the fourth-tier panels show the 8 mg/L tobramycin in monotherapy and combinations with ciprofloxacin. The solid lines and symbols represent the total population (Pop_TOT_) on drug-free agar plates and the hollow symbols with dashed and dotted lines are the tobramycin- (Pop_TOB_) and ciprofloxacin-less susceptible (Pop_CIP_) populations, respectively. All lines are mechanism-based model fits.

The time-course profiles of bacterial density as determined on antibiotic-free and antibiotic-containing plates for this combination are presented in Figure 4 for all four strains. The time-course profiles for the tobramycin and ciprofloxacin monotherapy arms of the combination studies with PAO1 and PAO∆*mutS* were in agreement with those from the monotherapy studies (Figure 2). Against PAO1, combinations with each of the three tobramycin concentrations and 1 mg/L ciprofloxacin exhibited synergy (>2 log_10_ CFU/mL killing compared to the most active monotherapy), whilst 4 mg/L ciprofloxacin in monotherapy was sufficient to suppress bacterial counts at 48 h. Notably, among all combinations, only the 1 and 4 mg/L tobramycin with 0.125 mg/L ciprofloxacin resulted in the emergence of less-susceptible populations to tobramycin (≤6.5 log_10_ CFU/mL), and ciprofloxacin (≤2.7 log_10_ CFU/mL).

#### 3.2.1. Antibacterial Effect of Tobramycin and Ciprofloxacin against the Three Hypermutator Strains

Against PAO∆*mutS*, all tobramycin concentrations were synergistic in combination with 1 and 4 mg/L ciprofloxacin. This strain exhibited less-susceptible populations to tobramycin for all treatments; however they were suppressed to values below that of the growth control for all combinations with 1 and 4 mg/L ciprofloxacin. No ciprofloxacin less-susceptible populations were observed for 4 and 8 mg/L tobramycin with 1 mg/L ciprofloxacin, or 8 mg/L tobramycin with 4 mg/L ciprofloxacin. Thus, the combination resulted in synergistic bacterial killing and the suppression of less-susceptible populations of PAO∆*mutS*. These results are in agreement with a previous study in a murine model that found the tobramycin and ciprofloxacin combination was synergistic against PAO∆*mutS* and resulted in reduced mortality and bacterial load without emergence of resistance [36].

The combination of these fast-acting antibiotics was additionally trialled against hypermutable clinical strains CW19 (mucoid) and CW44 (nonmucoid). Synergistic bacterial killing of CW19 was observed for 4 mg/L ciprofloxacin combinations with 4 and 8 mg/L tobramycin. Additionally, these concentrations allowed the suppression of less-susceptible populations to counts below those observed for the growth control, i.e., either no or very few colonies observed. For CW44, 1 mg/L ciprofloxacin combined with either 4 or 8 mg/L tobramycin, and 4 mg/L ciprofloxacin combinations with all tobramycin concentrations were sufficient to provide synergistic bacterial killing and suppress less-susceptible populations to below the growth control.

Therefore, the combination of tobramycin and ciprofloxacin appears promising against hypermutable *P. aeruginosa*, including clinical mucoid and nonmucoid strains, due to the synergistic antibacterial activity and suppression of less-susceptible populations.

#### 3.2.2. Mechanism-Based Mathematical Modelling of the Tobramycin and Ciprofloxacin Combination

MBM was utilised to characterise the time-course of bacterial killing and regrowth of the total and less-susceptible populations for all four strains exposed to tobramycin and ciprofloxacin in monotherapy and combination therapy (Figure 1). The coefficient of correlation for the observed vs. individual fitted viable counts for all strains was on average 0.92 (Figure S2). The MBM including both subpopulation and mechanistic synergy yielded unbiased and precise curve fits of the total populations for all strains (Figure 4). The parameter estimates are reported in Table 2. The MBM only required a single KC_50_ for each antibiotic to best describe the data, except for the PAO∆*mutS* that required different KC_50,TOB_ for the different subpopulations. Mechanistic synergy was incorporated for the tobramycin-resistant and ciprofloxacin-intermediate population, where the ciprofloxacin concentration required for half-maximal killing (KC_50,CIP_) was decreased by on average 5.9-fold in the presence of 8 mg/L tobramycin for all strains. The MBM for the non-hypermutable PAO1 required a Hill coefficient for tobramycin to achieve unbiased population fits. The MBM-estimated fractions of the less-susceptible populations able to grow on tobramycin- and ciprofloxacin-containing agar plates were on average 1.8 and 3.2 log_10_ higher, respectively, for PAO∆*mutS* than PAO1, in agreement with the impact of hypermutator phenotype on resistance emergence. The main mechanisms for aminoglycoside resistance are upregulation of the MexXY-OprM efflux pump, target site modifications and inactivation via enzymes [37,38]. For fluoroquinolones, several efflux pumps and target site mutations are the primary mechanisms of resistance [38]. These different mechanisms of resistance of the two antibiotics likely contributed to the subpopulation synergy identified for this combination. The mechanistic synergy described in the MBM was likely at least in part representative of tobramycin disrupting the outer membrane of *P. aeruginosa* as reported previously [25,39], enhancing the penetration of ciprofloxacin to its target site. Overall, the tobramycin and ciprofloxacin combination was found to be promising to combat hypermutable *P. aeruginosa*.

## 4. Conclusions

The current study systematically compared the antibacterial effects of monotherapy with a range of antibiotics having different mechanisms of action; we examined the time-course of bacterial counts and emergence of resistant populations of non-hypermutable and hypermutable *P. aeruginosa*. In addition we assessed the tobramycin and ciprofloxacin combination against four strains, including two hypermutable clinical strains. This is the first study to have characterised the time-course of bacterial killing, regrowth and emergence of resistance of hypermutable PAO∆*mutS* against multiple antibiotics over 48 h. Additionally, this is the first study to characterise the synergistic tobramycin plus ciprofloxacin combination against hypermutable clinical strains. We demonstrated that bacterial regrowth and emergence of less-susceptible populations over 48 h were generally more pronounced for PAO∆*mutS* than PAO1. Our results indicate that monotherapy with clinically relevant concentrations of commonly used antipseudomonal antibiotics is not a viable option to combat hypermutable *P. aeruginosa* due to the resulting emergence of resistant populations. Tobramycin plus ciprofloxacin was identified as a promising combination for synergistic killing and suppression of less-susceptible populations of hypermutable *P. aeruginosa* strains. MBM incorporating both subpopulations and mechanistic synergy well described bacterial killing, regrowth, and emergence of resistance. The MBM will be useful for the design of future studies in dynamic in vitro systems that are warranted to rationally optimise this combination against infections caused by these difficult to treat hypermutable strains.

## Figures and Tables

**Table 1:** Minimum inhibitory concentrations (MICs). The MIC values of the antibiotics used against hypermutable and non-hypermutable *Pseudomonas aeruginosa* strains.

	PAO1/PAOΔ*mutS* ^1^	CW19 ^2^	CW44 ^2^
aztreonam	4	-	-
ceftazidime	2	-	-
imipenem	2	-	-
meropenem	1	-	-
tobramycin	0.5	1	1
ciprofloxacin	0.125	0.5	0.19

^1^ Agar dilution MIC values using the Clinical and Laboratory Standards Institute (CLSI) method [11]. ^2^ Etest MIC values described previously [10]. PAO1: non-hypermutable *P. aeruginosa* wild-type reference strain; PAO∆*mutS*: hypermutable *P. aeruginosa* strain; CW19 and CW44: clinical hypermutable *P. aeruginosa* strains.

**Table 2:** Population mean (SE %) parameter estimates for the mechanism-based model (MBM) evaluation of static concentration time-kill (SCTK) experiments assessing the tobramycin (TOB) with ciprofloxacin (CIP) combination against non-hypermutable *P. aeruginosa* PAO1 and hypermutable PAO∆*mutS*, CW19, and CW44. All parameters described were required for the MBM to achieve unbiased and precise curve fits.

Parameter	Symbol (unit)	Population Mean Value (SE[%])			
		PAO1	PAOΔ*mutS*	CW19	CW44
***Bacterial growth and subpopulations***					
Initial inoculum	Log_10_ CFU_0_	7.09 (2.05%)	7.62 (1.71%)	7.87 (2.75%)	7.26 (1.39%)
Maximum population size	Log_10_ CFU_max_	9.33 (2.00%)	9.01 (1.79%)	9.00 (1.31%)	8.94 (1.01%)
Mean generation time (MGT)					
TOB^S^/CIP^S^	*1*/*k*_12,SS_ (min)	50.9 (4.55%) ^1^	55.9 (4.81%) ^1^	115 (6.26%) ^1^	124 (6.48%) ^1^
TOB^R^/CIP^I^	*1*/*k*_12,RI_ (min)	340 (13.1%)	254 (19.4%)	327 (4.96%)	141 (8.57%)
TOB^I^/CIP^R^	*1*/*k*_12,IR_ (min)	50.9 (4.55%) ^1^	55.9 (4.81%) ^1^	115 (6.26%) ^1^	124 (6.48%) ^1^
Log_10_ mutation frequencies					
TOB	Log_10_ MF_TOB_	−3.68 (5.43%)	−3.93 (6.52%)	−3.3 (16.6%)	−4.81 (4.01%)
CIP	Log_10_ MF_CIP_	−7.68 (4.04%)	−8.39 (4.60%)	−5.79 (12.7%)	−7.47 (5.12%)
***Killing by TOB***					
Maximum killing rate constant					
TOB^S^/CIP^S^	*K*_max,SS,TOB_ (h^−1^)	12.2 (11.2%)	10.8 (34.1%)	5.18 (36.1%)	3.26 (19.4%)
TOB^R^/CIP^I^	*K*_max,RI,TOB_ (h^−1^)	0.251 (22.9%)	0.305 (42.1%)	0.354 (15.1%)	0.60 (18.0%)
TOB^I^/CIP^R^	*K*_max,IR,TOB_ (h^−1^)	1.17 (15.1%)	0.367 (47.2%)	0.690 (62.5%)	5.02 (20.0%)
TOB concentration causing 50% of *K*_max,TOB_	KC_50,TOB_ (mg/L)	3.68 (21.5%)	2.11 (41.9%) ^2^	53.4 (10.1%)	18.5 (7.14%)
			7.33 (17.2%) ^3^		
			4.50 (14.9%) ^4^		
Hill coefficient for TOB	HILL_TOB_	0.790 (17.9%) ^5^			
***Killing by CIP***					
Maximum killing rate constant					
TOB^S^/CIP^S^	*K*_max,SS,CIP_ (h^−1^)	16.4 (9.95%)	17.1 (14.2%)	2.43 (11.7%)	5.11 (13.5%)
TOB^R^/CIP^I^	*K*_max,RI,CIP_ (h^−1^)	0.392 (20.5%)	0.307 (33.1%)	0.730 (17.5%)	1.14 (18.6%)
TOB^I^/CIP^R^	*K*_max,IR,CIP_ (h^−1^)	1.83 (13.6%)	0.812 (9.26%)	0.562 (13.4%)	0.226 (21.4%)
CIP concentration causing 50% of *K*_max,CIP_	KC_50,CIP_ (mg/L)	1.29 (24.3%)	1.09 (29.6%)	7.07 (41.3%)	8.30 (11.6%)
***Mechanistic synergy***					
Maximum fractional decrease of KC_50,CIP_ via mechanistic synergy^6^	*I*_max,SYN_	1 (fixed)	1 (fixed)	1 (fixed)	1 (fixed)
TOB concentration causing 50% of *I*_max,SYN_	IC_50,SYN_ (mg/L)	2.16 (11.0%)	1.10 (33.9%)	2.01 (10.9%)	1.74 (20.0%)
***Residual variability***					
SD of residual error on log_10_ scale					
Total population	SD_CFU_	0.303 (10.2%)	0.383 (12.1%)	0.296 (16.5%)	0.273 (10.1%)
Population on TOB plates	SD_CFU,TOB_	1.05 (24.1%)	0.401 (22.0%)	0.883 (14.1%)	0.197 (18.9%)
Population on CIP plates	SD_CFU,CIP_	3.64 (26.4%)	1.18 (14.4%)	0.586 (37.1%)	0.740 (31.1%)

^1^ Same mean parameter estimate was used for both the TOB^S^/CIP^S^ and TOB^I^/CIP^R^ populations; ^2^ TOB^S^/CIP^S^; ^3^ TOB^R^/CIP^I^; ^4^ TOB^I^/CIP^R^; ^5^ Hill function was required to describe data for PAO1; ^6^ Mechanistic synergy applied to the TOB^I^/CIP^R^ population, *I*_max,SYN_ was zero for the other populations.

## Supplementary Materials

The following are available online at https://www.mdpi.com/1999-4923/11/9/470/s1, Figure S1: The mean log_10_ CFU/mL and standard deviations (error bars) based on *n* = 3–4 replicates, except for ceftazidime where *n* = 2, for statistical analysis of key clinically achievable antibiotic concentrations against high inocula of PAO1 and PAOΔ*mutS*, Figure S2: Observed versus individual and population fitted viable counts for tobramycin and ciprofloxacin alone and in combinations against PAO1, PAO∆*mutS*, CW19, and CW44, and Table S1: The approximate unbound average steady-state plasma concentration of the maximum daily dose for the studied antibiotics.
