# Supplementary material for: Evaluation of Tobramycin and Ciprofloxacin as a Synergistic Combination Against Hypermutable Pseudomonas Aeruginosa Strains via Mechanism-Based Modelling
Source: Pharmaceutics. 2019 Sep 12;11(9):470. doi: 10.3390/pharmaceutics11090470 (PMC6781503; doi:10.3390/pharmaceutics11090470)
Supplement: Supplementary file 1 [file pharmaceutics-11-00470-s001.pdf]

pharmaceutics

Supplementary Materials: Evaluation of Tobramycin and Ciprofloxacin as a Synergistic Combination Against Hypermutable *Pseudomonas Aeruginosa* Strains via Mechanism-Based Modelling

Vanessa E. Rees, Jürgen B. Bulitta, Antonio Oliver, Roger L. Nation and Cornelia B. Landersdorfer

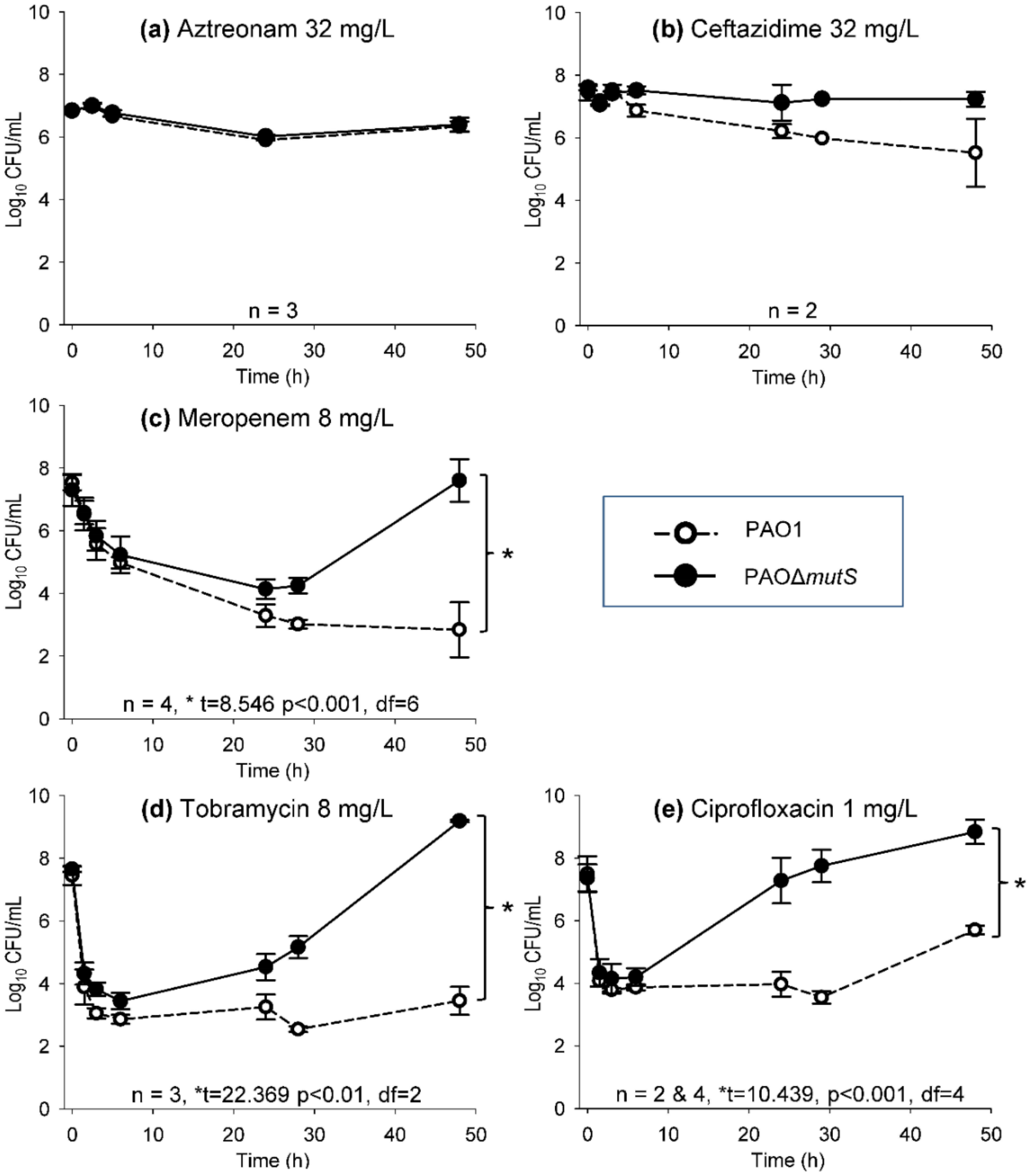

Figure S1. The mean $\log_{10}$ CFU/mL and standard deviations (error bars) based on n = 3–4 replicates, except for ceftazidime where n = 2, for statistical analysis of key clinically achievable antibiotic concentrations against high inocula of PAO1 and PAOΔmutS. The antibiotics studied were: (**a**), aztreonam 32 mg/L, (**b**), ceftazidime 32 mg/L, (**c**), meropenem 8 mg/L, (**d**), tobramycin 8 mg/L, and (**e**), ciprofloxacin 1 mg/L. The broken lines with hollow symbols represent PAO1 and the solid lines and symbols are PAOΔ*mutS*.

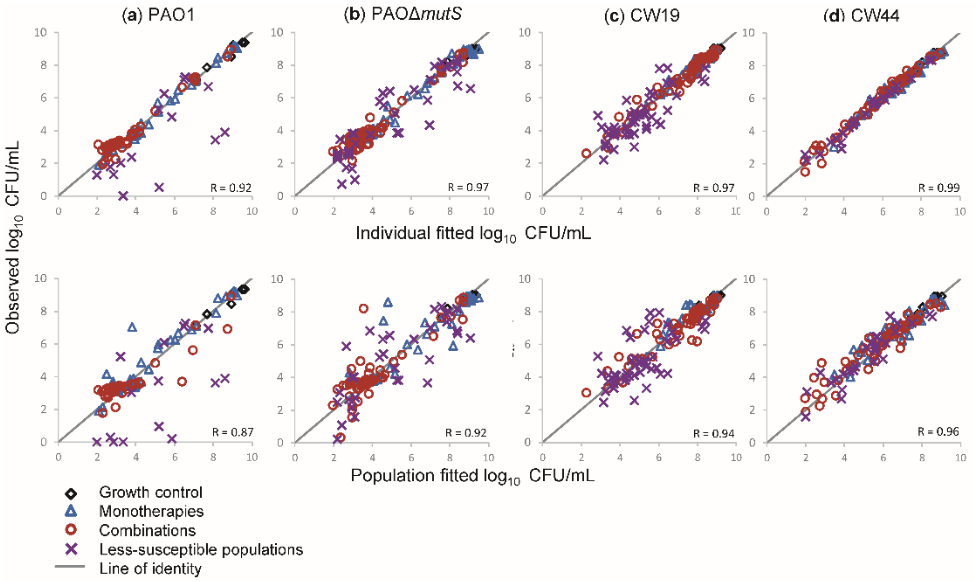

Figure S2. Observed versus individual and population fitted viable counts for tobramycin and ciprofloxacin alone and in combinations against PAO1, PAOΔ*mutS*, CW19 and CW44.

Table S1. The approximate unbound average steady-state plasma concentration of the maximum daily dose for the studied antibiotics.

Antibiotic	Maximum Daily Dose (mg)	~$fC_{ss,avg}$ (mg/L)	Reference
aztreonam	8000	26.7–35.2	[1]
ceftazidime	6000	33.8–41.4	[2]
imipenem	4000	12.2–18.8	[3,4]
meropenem	6000	15.0–15.4	[5,6]
tobramycin	700 [a]	3.1–3.9	[7,8]
ciprofloxacin	1200	0.96–1.4	[9,10]

[a] Based on 70 kg body weight, 10 mg/kg; $fC_{ss,avg}$, unbound average steady-state plasma concentration.

References

1. Vinks AA, van Rossem RN, Mathot RA, Heijerman HG, Mouton JW. Pharmacokinetics of aztreonam in healthy subjects and patients with cystic fibrosis and evaluation of dose-exposure relationships using monte carlo simulation. *Antimicrob Agents Chemother*. **2007**, *51*, 3049–3055.
2. Bulitta JB, Landersdorfer CB, Huttner SJ, Drusano GL, Kinzig M, Holzgrabe U, et al. Population pharmacokinetic comparison and pharmacodynamic breakpoints of ceftazidime in cystic fibrosis patients and healthy volunteers. *Antimicrob Agents Chemother*. **2010**, *54*, 1275–1282.
3. Jaruratanasirikul S, Raungsri N, Punyo J, Sriwiriyajan S. Pharmacokinetics of imipenem in healthy volunteers following administration by 2 h or 0.5 h infusion. *J Antimicrob Chemother*. **2005**, *56*, 1163–1165.
4. Sakka SG, Glauner AK, Bulitta JB, Kinzig-Schippers M, Pfister W, Drusano GL, et al. Population pharmacokinetics and pharmacodynamics of continuous versus short-term infusion of imipenem-cilastatin in critically ill patients in a randomized, controlled trial. *Antimicrob Agents Chemother*. **2007**, *51*, 3304–3310.
5. Krueger WA, Bulitta J, Kinzig-Schippers M, Landersdorfer C, Holzgrabe U, Naber KG, et al. Evaluation by monte carlo simulation of the pharmacokinetics of two doses of meropenem administered intermittently or as a continuous infusion in healthy volunteers. *Antimicrob Agents Chemother*. **2005**, *49*, 1881–1889.
6. Bui KQ, Ambrose PG, Nicolau DP, Lapin CD, Nightingale CH, Quintiliani R. Pharmacokinetics of high-dose meropenem in adult cystic fibrosis patients. *Chemother*. **2001**, *47*, 153–156.
7. Lee C, Walker SAN, Walker SE, Seto W, Simor A, Jeschke M. A prospective study evaluating tobramycin pharmacokinetics and optimal once daily dosing in burn patients. *Burns*. **2017**, *43*, 1766–1774.
8. Hennig S, Standing JF, Staatz CE, Thomson AH. Population pharmacokinetics of tobramycin in patients with and without cystic fibrosis. *Clin Pharmacokinet*. **2013**, *52*, 289–301.

9. Shah A, Lettieri J, Kaiser L, Echols R, Heller AH. Comparative pharmacokinetics and safety of ciprofloxacin 400 mg i.v. thrice daily versus 750 mg po twice daily. *J Antimicrob Chemother*. **1994**, *33*, 795–801.
10. Forrest A, Nix DE, Ballow CH, Goss TF, Birmingham MC, Schentag JJ. Pharmacodynamics of intravenous ciprofloxacin in seriously ill patients. *Antimicrob Agents Chemother*. **1993**, *37*, 1073–1081.
